# Supplementary material for: Assessing Institutional Stakeholders’ Perception and Limitations on Coping Strategies in Flooding Risk Management in West Africa
Source: Int J Environ Res Public Health. 2022 Jun 6;19(11):6933. doi: 10.3390/ijerph19116933 (PMC9180679; doi:10.3390/ijerph19116933)
Supplement: Supplementary file 1 [file ijerph-19-06933-s001.zip › ijerph-1692258-supplementary.pdf]

Stakeholder Interview Questions – LIRA 2030 Project

Project: Mitigating risks to flood-related waterborne diseases in Abidjan and Kampala

Interviewee:____	Date: ______________________________________
Interviewee affiliation: _________________________________	Site: __________________________________
Type of stakeholder: ___________________________________	Interviewer: ______________________________

Years of flooding experienced by the populations

Main geographical areas affected by flooding

Legal and institutional framework for the protection of the rights of vulnerable populations in case of flooding

----

Policy for the management of waterborne diseases in times of flooding

----

Status of stakeholders involved in flood management (who is involved in flood management?)

Level of collaboration between actors involved

Actions already carried out to support the populations or the focal point in the management of flood risks (morale, donation of medicine, food)

People most affected by flooding in your area

Gender mainstreaming during your flood management activities (the consideration of women, are there any of women, are there any special provisions for women)

Policy for the management of waterborne diseases in times of flooding

----

Flood risk prevention and response measures

Actions already carried out to support the populations or the focal point in the management of flood risks (morale, donation of medicine, food)
